# Early monocyte response following local ablation in hepatocellular carcinoma

**DOI:** 10.3389/fonc.2022.959987

**Published:** 2022-10-24

**Authors:** Melanie A. Kimm, Sophia Kästle, Matthias M. R. Stechele, Elif Öcal, Lisa Richter, Muzaffer R. Ümütlü, Regina Schinner, Osman Öcal, Lukas Salvermoser, Marianna Alunni-Fabbroni, Max Seidensticker, S. Nahum Goldberg, Jens Ricke, Moritz Wildgruber

**Affiliations:** ^1^ Department of Radiology, University Hospital, Ludwig-Maximilians-Universität München, Munich, Germany; ^2^ Core Facility Flow Cytometry, Biomedical Center Munich, Ludwig-Maximilians-Universität München, Planegg-Martinsried, Germany; ^3^ Goldyne Savad Institute of Gene Therapy, Hadassah Hebrew University Hospital, Jerusalem, Israel; ^4^ Laboratory for Minimally Invasive Tumor Therapies, Department of Radiology, Beth Israel Deaconess Medical Center, Harvard Medical School, Boston, MA, United States; ^5^ Division of Image-guided Therapy and Interventional Oncology, Department of Radiology, Hadassah Hebrew University Hospital, Jerusalem, Israel

**Keywords:** hepatocellular carcinoma, local ablation, brachytherapy, radiofrequency ablation, monocytes, flow cytometry

## Abstract

Local ablative therapies are established treatment modalities in the treatment of early- and intermediate-stage hepatocellular carcinoma (HCC). Systemic effects of local ablation on circulating immune cells may contribute to patients’ response. Depending on their activation, myeloid cells are able to trigger HCC progression as well as to support anti-tumor immunity. Certain priming of monocytes may already occur while still in the circulation. By using flow cytometry, we analyzed peripheral blood monocyte cell populations from a prospective clinical trial cohort of 21 HCC patients following interstitial brachytherapy (IBT) or radiofrequency ablation (RFA) and investigated alterations in the composition of monocyte subpopulations and monocytic myeloid-derived suppressor cells (mMDSCs) as well as receptors involved in orchestrating monocyte function. We discovered that mMDSC levels increased following both IBT and RFA in virtually all patients. Furthermore, we identified varying alterations in the level of monocyte subpopulations following radiation compared to RFA. (A) Liquid biopsy liquid biopsy of circulating monocytes in the future may provide information on the inflammatory response towards local ablation as part of an orchestrated immune response.

## Introduction

Liver cancer is the second leading cause of cancer death worldwide (1). Etiological causes of hepatocellular carcinoma (HCC) include chronic viral infections, high alcohol intake, and increasingly non-alcohol-related steatohepatitis (NASH), whereby the majority of HCC arises in the setting of chronic liver inflammation (2). Clinical decision-making in HCC is further dependent on staging systems that include tumor size and burden as well as liver function (3). The “Barcelona Clinic Liver Cancer” (BCLC) staging system is commonly used to link the prognostic stage of HCC to the best first-line treatment option. In early-stage HCC, curative surgical treatments, such as resection or transplantation, are first-line therapies (4). However, many patients do not fulfill the criteria for surgical treatment options, whereby image-guided local ablative therapies represent an alternative, either as definitive treatment or as a bridging option to transplantation (3).

Radiofrequency ablation (RFA) is a well-established method in interventional oncology and has been proven to be safe and effective for the treatment of early HCC (5, 6), but is limited with respect to a certain tumor size and tumor location. Interstitial brachytherapy (IBT) is a valuable alternative with deposition of an ionizing source (e.g., ^192^Iridium) within the tumor tissue instead of using external beam radiation, thereby limiting the injury of adjacent non-tumor tissue, which is particularly important in the setting of impaired liver function (7). IBT allows for safe and effective ablation even in patients with tumors larger than 3 cm, and who are not suitable for treatment by RFA or microwave ablation (MWA) (8). Notably, both local ablative therapies are able to induce effects on the local as well as on the systemic immune response (9, 10). Local effects are mainly related to the tumor microenvironment (TME), which is a heterogeneous composition of interacting tumor and non-tumor cells (such as fibroblasts and immune cells) and extracellular matrix components (11). Any change of this highly sensitive cell-to-cell communication mediated by internal or external alterations can support either pro- or anti-tumorigenic immune responses (12). Although it is still puzzling which cell–cell interactions, communication signals, and polarization events within TME of HCC will lead to an anti- or pro-tumorigenic effect, it is well established that myeloid-derived cells such as monocytes, macrophages, and dendritic cells are key players within this theater (13).

In the liver, tissue-resident macrophages (Kupffer cells) play a central role in maintaining liver homeostasis and upon acute liver injury become activated and differentiate into immune-activating or immune-suppressive phagocytes (14). In addition, myeloid cell populations from the peripheral blood [monocytes and myeloid-derived suppressor cells (MDSCs)] are recruited to the site of injury (15, 16). When entering the tissue, monocytes differentiate into macrophages and the TME orchestrates the polarization into certain phenotypes. Whether different ablative treatments induce differences in pre-priming of peripheral blood monocytes is not yet elucidated. This pre-priming of circulating immune cell populations may serve as a predictive or prognostic biomarker for response to targeted treatment or may indicate severity of tissue injury. Recent studies have identified lymphocyte-to-monocyte ratio (LMR), neutrophil-to-monocyte ratio (NMR), and neutrophil-to-lymphocyte ratio (NLR) as potential prognostic markers for survival of HCC patients (17–21). Yet, little is known about possible differences in peripheral blood myeloid cell subpopulations following local ablative therapies, such as IBT and RFA.

Thus, in this study, we aimed to investigate priming of circulating monocytes following local ablation in early- and intermediate-stage HCC.

## Material and methods

### Patients and study design

Patients were recruited in two prospective clinical trials investigating the image-guided local ablation of early- and intermediate-stage HCC. The analysis consists of 21 patients with HCC. The ESTIMATE trial investigates the effects of IBT, from which 12 patients were recruited. In order to compare the effects after local radiation to RFA, nine patients were included from the THIAMAT trial. An overview of the patients’ clinical characteristics is shown in **Table 1**. Blood samples were obtained at baseline on the day before local ablation as well as 24–48 h post-IBT/RFA.

**Table 1:** Clinical characteristics of the study cohort.

		IBT (*n* = 12)	RFA (*n* = 9)	*p*-value
Sex	Female	3	1	0.6030^a^
	Male	9	8	
Age at therapy start^#^		71.50 (7.50)	70.00 (14.00)	1.0000^b^
Cirrhosis		10/12	8/9	1.0000^a^
Child pugh score	A	8	7	0.3865^c^
	B	2	1	
NASH		1/8	2/8	1.0000^a^
Diabetes mellitus Type II		7/12	5/9	1.0000^a^
High alcohol intake		5/8	4/7	1.0000^a^
Viral hepatitis		2/8	1/8	1.0000^a^
Mixed etiology		2/8	2/8	1.0000^a^
Maximal tumor diameter [mm]^*^		34.73 (± 14.56)	31.93 (± 14.61)	0.6691^d^
⌀ amount of tumors^#^		1.00 (1.00)	2.00 (1.00)	0.0091^b^
BCLC score	BCLC 0/A	2/6	0/3	0.4753^c^
	BCLC B/C	2/2	2/4	
AFP [ng/ml]^#^		16.05 (412.85)	8.55 (13.80)	0.9079^b^
Serum albumin [g/dl]^*^		3.92 (± 0.61)	3.87 (± 0.43)	0.8370^d^
Total bilirubin [mg/dl]^#^		0.70 (0.90)	0.80 (0.60)	0.4920^b^
Platelet count [G/L]^#^		130.50 (56.00)	114.00 (75.00)	0.9716^b^
Therapy outcome (Responder/Non-Responder)		11/1	7/2	0.5534^a^

The size of tumor lesions was measured as maximal tumor diameter of the largest lesion. AFP, a-fetoprotein; BCLC, Barcelona Clinic Liver Cancer staging system; NASH, non-alcoholic steatohepatitis. ^#^median (IQR), ^*^mean ( ± SD), a, Fisher’s exact test, b, Mann–Whitney U-test, c, Chi-square test, d, t-test.

### Ethics

The studies were approved by the local ethics commission of the university hospital (LMU München, Munich, Germany), with German clinical trial register numbers DRKS 00010587 (ESTIMATE) and DRKS 00010560 (THIAMAT). All study protocols were conducted in accordance with the Declaration of Helsinki. Informed consent of each participant was obtained prior to enrollment.

### Patient response assessment

Patients were stratified into responders versus non-responders based on previously published criteria for HCC disease stages (4) and eligibility for curative versus palliative treatments in case of progression. Accordingly, responders were defined as patients showing complete remission for a minimum of 6 months following therapy. Any recurrence seen within 6 months post-therapy or tumor appearance in between a total follow-up period of 24 months greater than 3 cm or >3 tumor lesions classified the patient as a non-responder.

### Leukocyte ratios

LMR and NMR were computed as absolute numbers of lymphocytes and neutrophils, respectively, divided by monocytes. NLR was computed as absolute number of neutrophils divided by lymphocytes. Pre- and post-therapy LMR, NMR, and NLR were calculated from absolute numbers of monocytes, lymphocytes, and neutrophils (G/µl). In total, from all 12 ESTIMATE patients and from 7/9 THIAMAT patients, cell numbers were available and analyzed.

### PBMC collection and flow cytometry analysis

**Figure 1:**
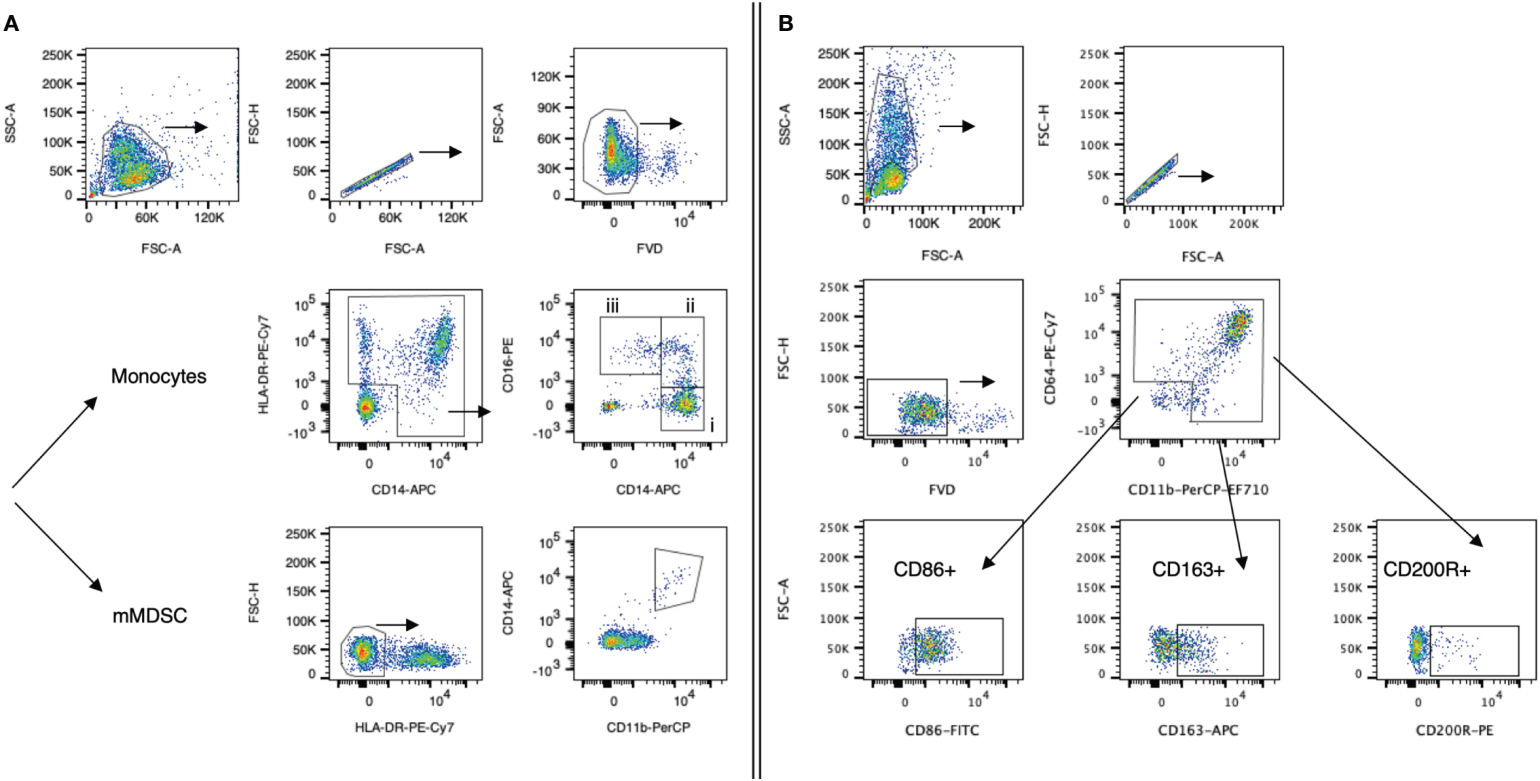
Gating strategies for myeloid cell populations. Representative dot plots from one HCC patient. **(A)** Panel 1: Monocytes were defined as HLA-DR+CD14+ cells. Monocyte subsets (classical, intermediate, non-classical; all presented as % frequency of monocytes) were further defined by their expression of CD14 and CD16: (i) classical monocytes (CD14++CD16−), (ii) intermediate monocyte (CD14++CD16+), and (iii) non-classical monocytes (CD14+CD16++). Monocytic MDSCs (mMDSCs) were defined as HLA-DR-CD11b+CD14+ cells (% of live cells). Cells were fixed prior to staining. **(B)** Panel 2: CD11b+CD64+ monocytes were further analyzed for CD86, CD163, and CD200R marker expression. Cells were not fixed prior to staining. Following staining, cells of both panels were fixed with 4% PFA and subsequently analyzed. FVD, Fixable Viability Dye-eFluor^®^780.

PBMCs were isolated using a Ficoll-Paque density gradient (Cytiva, Uppsala, Sweden) and cryopreserved until analyzed. The following monoclonal antibodies specific for human antigens were used: anti-CD14-APC (63D3), anti-CD16-PE (B73.1), anti-CD64-PE-Cy7 (10.1), anti-CD86-FITC (BU63), anti-CD163-APC (GHI/61), anti-CD200R-PE (OX-108), anti-HLA-DR-PE-Cy7 (L243), anti-IgG1 (MOPC-21) (all from BioLegend, San Diego, CA, USA), anti-CD11b-PerCP-eFluor^®^710 (ICRF44), and Fixable Viability Dye-eFluor^®^780 (all from ThermoFisher Scientific, Waltham, MA, USA). In brief, cells were thawed and resuspended in staining buffer (1× PBS/3% FBS). Staining against surface antigens only (panel 2) or surface antigens and cytoplasmic protein (panel 1, data for intracellular staining not shown) was performed for 30 min at 4°C in the dark. Staining against cytoplasmic protein was performed after cell fixation with 2% PFA. Cells were analyzed on the flow cytometer FACSCanto (BD Biosciences, Immune Cytometry Systems, San Jose, CA, USA), and data were analyzed using FlowJo software version 10 (BD Life Sciences, Ashland, OR, USA). The gates were set based on Fluorescence-minus-one (FMO) and IgG control antibody staining, and the number in each gate represents the percentage of cells. Gating strategy for monocyte subsets and mMDSC (panel 1) is shown in **Figure 1A**, and gating strategy for the myeloid polarization markers (panel 2) is shown in **Figure 1B**. Monocyte subsets, CD86+, D163+, and CD200R+ cells were presented as percentage of monocytes; mMDSCs were presented as percentage of viable cells. Monocyte gating strategies and nomenclature were applied following the principles by Ziegler-Heitbrock et al. (22, 23); those for mMDSCs were based on Gabrilovich et al. (24, 25).

### Histology

Tumor biopsies were fixed in 3.7% neutral-buffered formaldehyde and embedded in paraffin according to standard protocols. Two-micrometer sections were prepared, and morphology was visualized by standard H&E staining. For immunohistochemistry, the activity of the endogenous peroxidase was blocked with 1% hydrogen peroxide, and after antigen retrieval (citric acid buffer, pH 6) at 100°C, sections were incubated with anti-CD68 (dilution 1:250, clone KP1, ThermoFisher Scientific, Waltham, MA, USA), anti-CD86 (dilution 1:75, clone E2G8P, Cell Signaling Technology, Beverly, CA, USA), and anti-CD163 antibody (dilution 1:250, clone D6U1J, Cell Signaling Technology, Beverly, CA, USA), respectively. This was followed by incubation with EnVision™+Dual Link System-HRP (Dako, Carpinteria, CA, USA). Diaminobenzidine (Cell Signaling Technology, Beverly, CA, USA) was used as a chromogen. Sections were counterstained with 1% Mayer’s hematoxylin. Slides were analyzed using a Leica dm2500 microscope equipped with LAS version 4 software (Leica, Germany).

### Statistical analysis

Statistical analysis was performed using GraphPad Prism (version 9, GraphPad Software, San Diego, CA, USA) and SAS (version 9.4, SAS Institute Inc., Cary, NC, USA). Normality distribution was determined by the Shapiro–Wilk test. Paired data were analyzed using paired *t*-test or Wilcoxon test. We calculated intraindividual differences delta to take care of the dependencies in the data (pre- and post-treatment values of the same patient) and compared the independent deltas between the two different cohorts using *t*-test or Mann**–**Whitney *U*-test, depending on normality of data. For analysis of clinical and demographic data, Fisher’s exact test, Mann–Whitney *U*-test, *t*-test, and chi-square test were used in dependency of the normal distribution. Non-normally distributed data are presented as median with interquartile range (IQR), and normally distributed data are presented as mean with standard deviation (SD). A *p* **-**value < 0.05 was considered significant.

## Results

### Patient characteristics of the study cohort

All patients were recruited through the liver clinics in a tertiary care/liver transplant center and diagnosed with HCC based on radiological criteria and biopsy. Patient characteristics and liver function tests at treatment baseline are summarized in **Table 1**. No significant differences were observed between the two patient cohorts for most of the listed parameters.

**Figure 2:**
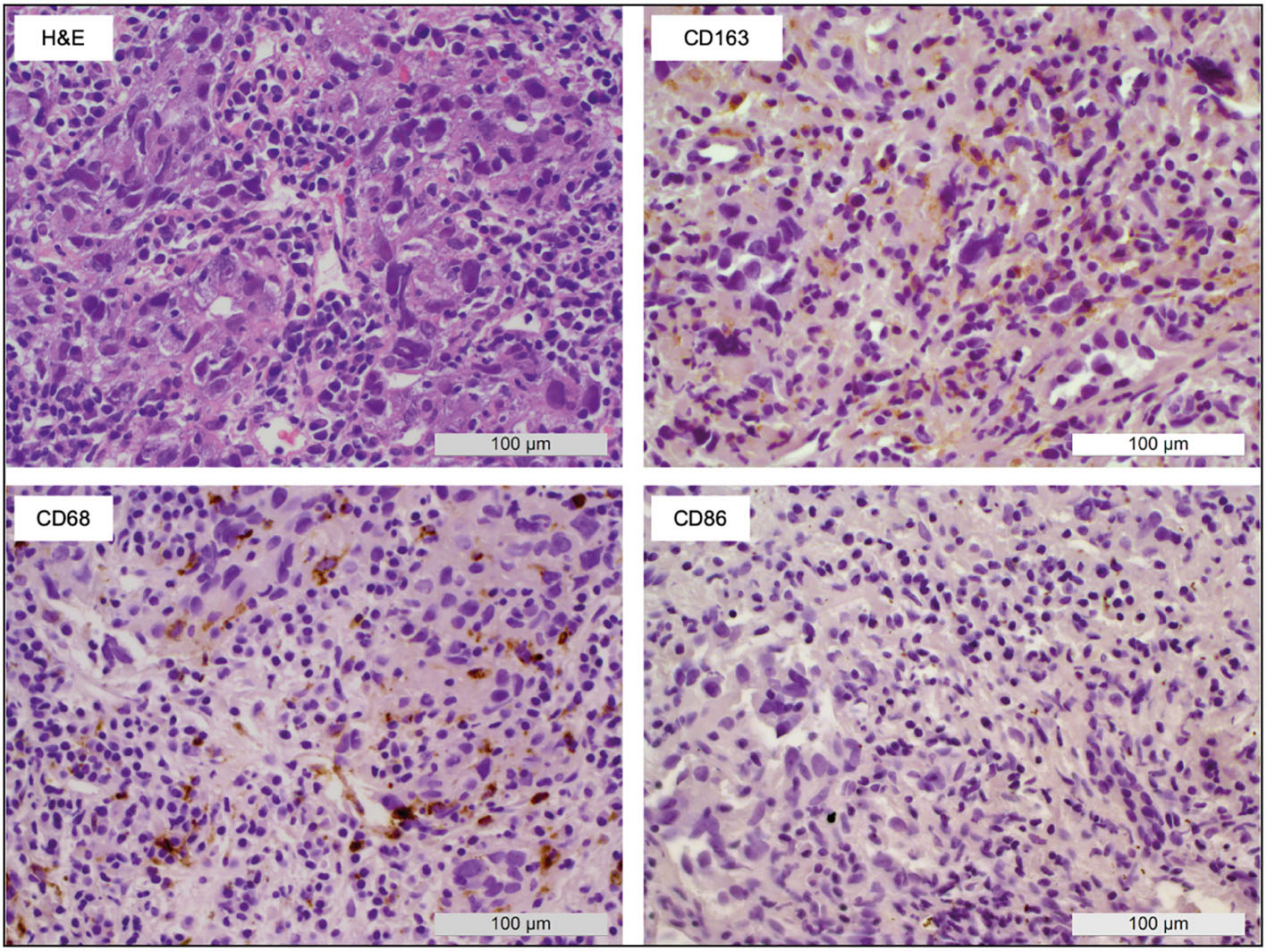
Macrophage polarization within HCC biopsies. A representative sample shows the appearance of macrophages in tumor tissue obtained before local ablation. H&E staining as well as CD68 (pan macrophage), CD86 (M1), and CD163 (M2) IHC allowed the identification of large areas with M2-type macrophages (CD163+) whereas M1-type macrophages (CD86+) were almost completely absent.

Tumor-associated macrophage staining (CD68) of tumor biopsies of nine patients obtained at baseline before local ablation revealed tumor regions with CD163+ cells in all patients. This denoted an inflamed liver microenvironment and immunosuppressive M2-phenotypic TME. In turn, CD86 staining, indicative of M1 polarization of macrophages, was almost completely absent (**Figure 2**).

### Alterations in leukocyte populations and ratios following IBT and RFA

**Figure 3:**
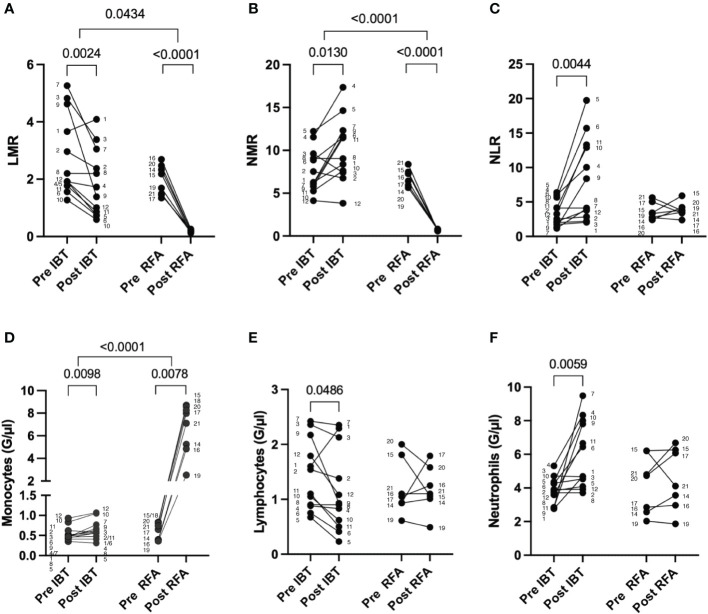
Leukocyte changes following different local ablation treatments. Pre and post analysis of **(A)** LMR, **(B)** NMR, **(C)** NLR, **(D)** monocytes, **(E)** lymphocytes, and **(F)** neutrophils. Differential blood values from 12 IBT- and 7 RFA-treated patients were analyzed. Each dot represents an individual patient. Numbers next to dots represent patient IDs and ensure assignment of pre and post values. Data were analyzed using paired *t*-test (IBT: **B, C, E**, and F; RFA: **A–C, E, F**) or Wilcoxon-test (IBT: **A, D**; RFA: **D**). Intraindividual differences were analyzed using unpaired *t*-test **(A, B, D–F)** or Mann–Whitney *U*-test **(C)**. *p*-values < 0.05 indicate statistical significance.

First, we investigated peripheral blood LMR, NMR, and NLR following local ablative therapy IBT and RFA (**Figure 3**). Both treatments resulted in significant changes in LMR and NMR, IBT additionally in NLR. Following IBT, LMR decreased from 2.06 pre-therapy to 1.85 post-therapy (*p* = 0.0024) (**Figure 3A**) while NMR increased from 7.70 to 10.16 (*p* = 0.0130) (**Figure 3B**) and NLR increased from 3.38 to 8.26 following treatment (*p* = 0.0044) (**Figure 3C**). Strikingly, following RFA, both LMR and NMR dropped close to zero in all patients analyzed, indicating a tremendous increase in monocytes. LMR values decreased post-RFA from 1.95 to 0.19 (*p* < 0.0001) (**Figure 3A**), and NMR decreased from 6.60 to 0.70 (*p* < 0.0001) (**Figure 3B**). No significant changes were obtained for NLR in patients following RFA (**Figure 3C**). Detailed specifications are listed in **Supplementary Table 1**. These results point to the different mode of action between IBT and RFA.

Looking into the distribution of the different leukocyte populations in more detail, we found an increase in monocyte and neutrophil numbers following IBT, while lymphocyte numbers decreased (**Figures 3D–F**). In contrast, following RFA, only peripheral blood monocytes increased in numbers (**Figure 3D**), whereas lymphocytes and neutrophils showed no significant changes (**Figures 3E, F**).

### Interventional therapy-related dynamics of peripheral blood myeloid cell populations

**Figure 4:**
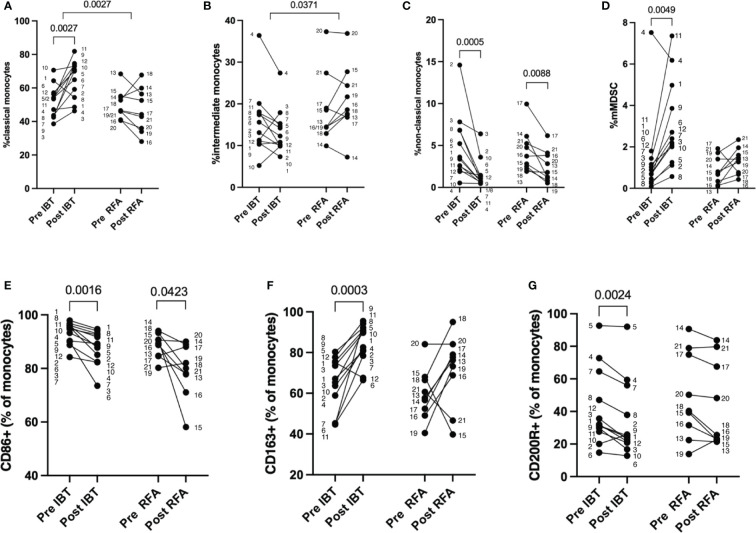
Therapy-specific alterations in monocyte cell populations. Percentage of myeloid cells as measured by flow cytometry. Pre and post analysis of 12 IBT- and 9 RFA-treated patients. **(A)** Classical monocytes (CD14++CD16−), **(B)** intermediate monocytes (CD14++CD16+), **(C)** non-classical monocytes (CD14+CD16++), **(D)** mMDSC, **(E)** CD86+, **(F)** CD163+, and **(G)** CD200R+ monocytes (shown as percentage of monocytes). Each dot represents an individual patient. Numbers next to dots represent patient IDs and ensure assignment of pre and post values. Data were analyzed using paired *t*-test (IBT: **A, F**; RFA: **A, C–F**) or Wilcoxon test (IBT: **B–E, G**; RFA: **B, G**). Intraindividual differences were analyzed using unpaired *t*-test **(A, B, D, E)** or Mann–Whitney *U*-test **(C, F, G)**. *p*-values < 0.05 indicate statistical significance.

To gain more insight into treatment-related differences in monocyte subpopulations between radiation-based IBT and heat-based RFA, we analyzed the peripheral blood of 12 IBT- and 9 RFA-treated patients pre- and post-treatment (**Figure 4**) using flow cytometry. In addition to the three major subpopulations—classical, intermediate, and non-classical monocytes (**Figures 4A–C**)—we identified mMDSC (**Figure 4D**) and analyzed the expression of CD86, CD163, and CD200R indicative for monocyte activation and differentiation (**Figures 4E–G**). Healthy donor data of three non-matched individuals showed no difference for monocyte subpopulations and mMDSC at baseline (**Supplementary Figure 1**).

Compared to baseline, we found significantly increased fractions of classical monocytes in 11 out of 12 patients following IBT (52% pre vs. 66% post, *p* = 0.0027) (**Figure 4A**, **Supplementary Table 1**). Only in patient no. 1 did classical monocyte levels decrease post-IBT; still, the patient responded well to therapy. The most relevant clinical parameter that differed compared to other patients was a chronic hepatitis B infection.

Patients treated with RFA were heterogeneous in regard to classical monocyte proportion (**Figure 4A**). Only two patients showed an increase in the proportion of classical monocytes following RFA (patient no. 14 and patient no. 18), but no relevant clinical parameters could be clearly correlated to this observation. Furthermore, we determined intraindividual differences (delta) for each patient cohort and analyzed possible differences between the two treatment options. With regard to classical monocyte percentages, we observed significant differences between the two treatments (*p* = 0.0027). Looking at intermediate monocyte frequencies, we saw no significant changes compared to baseline neither for IBT- nor RFA-treated patients (14% pre vs. 12% post, *p* = 0.1294 and 15% pre vs. 21% post, *p* = 0.2031, respectively) (**Figure 4B**, **Supplementary Table 1**). Yet, the comparison between treatment modalities revealed significant difference concerning the proportion of intermediate monocyte population (*p* = 0.0371). While IBT led to decreased intermediate monocyte fractions, they were overall increasing following RFA (**Supplementary Table 1**). Interestingly, non-classical monocyte levels significantly decreased unrelated to one or the other treatment (IBT: 4% pre vs. 1% post, *p* = 0.0005 and RFA: 5% pre vs. 3% post, *p* = 0.0088) (**Figure 4C**, **Supplementary Table 1**) even though the effect was more pronounced following IBT.

Next, we analyzed proportions of peripheral blood mMDSC and detected in virtually all IBT-treated patients significantly increased mMDSC proportions (1% pre- vs. 2% post-therapy, *p* = 0.0049) (**Figure 4D**, **Supplementary Table 1**). Only in one patient (patient no. 4) did we observe a decrease in mMDSC following IBT. This patient represents the only non-responder in the IBT-treated patient cohort. Following RFA, we found in 8 patients increasing levels of mMDSC regardless of the response status. One patient had decreasing mMDSC proportion (patient no. 17), and one patient (patient no. 19) showed equal percentages pre- and post-therapy. In comparison to the non-responding patient following IBT, both non-responding patients of the RFA-treated patient cohort showed increasing mMDSC frequencies (all RFA-treated patients: 0.87 pre vs. 1.32 post, *p* = 0.1081). Compared to the other patients, there was no clinically evident cause explaining the decreased mMDSC.

Furthermore, we investigated possible variance in expression of CD86, CD163, and CD200R pre- and post-treatment (**Figures 4E–G**). The rate of CD86+ monocytes showed a tendency to decrease in both patient cohorts, but not reaching significant differences (IBT: 86% pre vs. 81% post, *p* = 0.0772; RFA: 82% pre vs. 72% post, *p* = 0.0508) (**Figure 3E**, **Supplementary Table 1**). At the same time, we observed a significant increase in scavenger receptor CD163-expressing monocyte proportions following IBT (55% pre vs. 80% post, *p* < 0.0001), and even though not significant, we observed a gain in CD163+ monocyte fraction following RFA (49% pre vs. 62% post, *p* = 0.1298) (**Figure 4F**, **Supplementary Table 1**). Only for one IBT-treated patient (patient no. 12) did we observe a decrease in CD163+ monocyte percentages. This result may be explained by an underlying hemochromatosis with a mutation in the HFE gene. Two RFA-treated patients showed decreased CD163+ proportions. Patient no. 15 was classified as a non-responder; patient no. 21 was the only HBV-positive patient within the RFA-treated cohort. When looking at the dynamics of CD200R+ monocyte fractions, we discovered certain differences. Following IBT, CD200R+ monocyte levels significantly decreased (16% pre vs. 10% post, *p* = 0.0425) (**Figure 4G**, **Supplementary Table 1**). Only two IBT-treated patients had increasing CD200R+ proportions (patient no. 2 and patient no. 5). We could not identify relevant clinical parameters for patient no. 2 explaining an increase in CD200R+. Patient no. 5, however, has an underlying autoimmune hepatitis. In comparison, we could not detect significant differences in CD200R expression levels in monocytes of RFA-treated patients (**Figure 4G**) (**Supplementary Table 1**).

For none of the three markers analyzed did we find a significant difference between the two types of treatment (**Figures 4E–G**).

## Discussion

Liver cancer is an inflammation-associated tumor that develops on the ground of injured liver tissue—cirrhosis. The inflammatory reaction originally intended for tissue repair usually develops towards a chronic condition, which is able to promote tumorigenesis and growth (26). Inflammation is considered a hallmark of cancer and the innate immune response is a major player in orchestrating both the local and systemic response (27). Furthermore, myeloid cells play a critical role in the resolution phase of inflammation (28) and activating myeloid cell populations following therapy could affect patients’ response, resulting in positive or negative abscopal effects. The TME plays a decisive role, and its composition mainly determines whether a pro- or anti-tumorigenic environment dominates the scene. Herein, tumor-associated macrophages are one of the most abundant cell types in the TME. They mature from peripheral circulating monocytes and, depending on prevalent mediators, differentiate into pro-inflammatory M1 or anti-inflammatory M2 macrophages. Additionally, under pathological conditions, tissue-resident Kupffer cells become activated and gain either M1 or M2 function (29, 30).

Local ablative therapies have proven effective in the treatment of primary liver tumors and their combination with immunotherapies is currently an increasing focus of research. IBT as locally effective radiotherapy and heat-induced tumor destruction by RFA or microwave ablation represent common forms of interventional tumor therapy (31). Both IBT and RFA are able to induce immunogenic cell death leading to increased antigen presentation and the release of damage-associated molecular patterns (DAMP) due to cell necrosis, which may finally result in an anti-tumorigenic response (32–35). In both types of therapy, next to the destruction of tumor tissue, adjacent liver tissue and vasculature is affected and potentially injured. The associated inflammatory reaction is of interest, as it may contribute to the patient’s therapy response beyond the initial tumor destruction. This raises several questions: Is the immune reaction restricted locally or is it possible to detect systemic effects within the peripheral blood? Are there differences in the systemic immune reaction depending on the type of interventional therapy applied? To answer these questions, we analyzed the dynamics of circulating blood monocytes and the expression of monocyte function-related markers using flow cytometry in a cohort of 21 HCC patients treated with either IBT or RFA. Due to the applied study protocols and the prospective character of the studies, our analysis does not include data from patients with the same degree of liver cirrhosis, but without cancer. This is a clear limitation of the herein presented data and future studies shall address this issue in more detail.

We found significant changes in leukocyte ratios LMR, NMR, and NLR that indicate systemic effects following ablation therapy. Furthermore, we detected changes in monocyte proportions, monocyte subpopulations, mMDSCs, and distinct monocyte markers at 24 to 48 h after the respective therapy. The changes occurred independent of the patients’ response status, but differed regarding the treatment modality. Noteworthy, the analysis revealed an increase in absolute monocyte numbers that was significantly higher following RFA, compared to IBT treatment. This could indicate the degree of early injury, which initially is more pronounced in RFA compared to IBT, where cell death and necrosis develop in due course. In addition to monocytosis, we noted lymphopenia and neutrophilia following IBT, but not RFA, representing an inflammatory leukogram that is caused by IBT-induced necrosis and thus related to the induction of immunogenic cell death. Hence, differences in leukocyte populations may hint to temporal differences in wound healing phases following either one of the ablative treatments. In addition, RFA and IBT clearly induce different systemic immune reactions, which might correlate to the degree and mode of tissue injury and the associated inflammatory response.

With respect to the different monocyte subsets, we observed a substantial decrease in non-classical monocytes, no matter which treatment modality was applied. A drop in non-classical monocytes may indicate their migration to the liver, and since non-classical monocytes are associated with wound healing (36, 37), they might be recruited for tissue repair following tumor ablation. Even though classical monocytes are the predominant population, which upon tissue injury is recruited from the blood to the site of injury (38), we noted increased proportions of classical monocytes only following IBT, not after RFA. Classical monocytes have an important function in the initiation and progression of the inflammatory response (39), and differences in the appearance of classical monocyte proportions could be related to radiation-induced cell death, which occurs over an extended time frame of at least several days, while RFA induced cell death is an immediate event. Furthermore, we noted that changes within intermediate monocyte subsets differed significantly between IBT- and RFA-treated patients. Changes in peripheral monocyte subsets may also indicate how fast ablation-induced inflammation resolves (39, 40). A clear limitation of this study is that only one time point after local ablation was sampled. Kinetic studies will be needed to obtain a more precise picture on the recruitment and re-storage of individual monocyte subsets following the different interventional therapies. Such studies are also necessary to better understand how the wound healing phases temporally differ depending on the ablation mode.

The increase in mMDSC fractions observed in both patient cohorts also suggests a treatment-related effect, although the increase in mMDSC was only significant following IBT. Again, this might be the result of the different modes of action and may indicate a stronger inflammatory response post-IBT. Monocytic MDSCs are only found under pathological conditions (40–42), and as they are able to enhance or restore immune reactions, their function is a double-edged sword (43). mMDSC accumulation is commonly linked to a worse prognosis of patients in a wide range of cancers (44). However, given that virtually all treated patients had a good response to ablation (18/21), defined as no recurrence of the disease within 6 months after treatment, we conclude that the mMDSC signature in the peripheral blood that we observed at the given time point is treatment- rather than response-related. Immunosuppression is a critical part during wound healing (45), and the recruitment of mMDSCs following ablative therapy may indicate the healing process. To delineate the role of mMDSC following local ablation, more mechanistic studies, including animal models of local ablation, would be needed. The descriptive character of our study is a clear limitation and more studies are needed to reveal the role of mMDSC following ablative therapies in HCC.

The observation that the proportion of CD163+ monocytes was increased independent of the type of treatment applied was remarkable. CD163 is a scavenger receptor, physiologically involved in the clearance of hemoglobin after red blood cell lysis (46). Changes in CD163+ monocyte proportion could indicate complications, technically as well as those that are hemolysis-related. Hemolysis was described in patients following thermal injury, and the circumstance that hemolysis can lead to acute kidney failure makes CD163 an interesting marker for monitoring patients after ablative treatments. Further studies with higher patient numbers are needed to better understand why radiation-induced injury caused significantly increased CD163+ monocyte fractions whereas thermal-induced injury did not. The tendency of CD86+ monocyte fractions to decrease following ablative treatment suggests a functional shift from an inflammatory environment towards an anti-inflammatory one within the peripheral blood. In complex with CD80, the CD86 receptor interacts with CD28 on T lymphocytes and is part of the full activation of CD4+ T cells. Finally, this leads to CTLA-4 upregulation that competes with CD28 for CD80/86 binding, resulting in the termination of T-cell stimulation. Furthermore, CD86 is a marker for APC activation (47) and CD86 receptor downregulation can lead to an anti-inflammatory and immune-regulatory phenotype (48) and may indicate the presence of a wound healing phase following ablation therapy. With regard to CD200R+ monocytes, we detected decreased fractions following treatment with IBT, but no relevant changes following RFA. CD200–CD200R is a known immunoregulatory checkpoint axis with CD200R mainly expressed on myeloid cells and T cells. It is one key player in regulating immune homeostasis, especially in maintaining immune tolerance (49, 50). We identified in one patient with autoimmune hepatitis (AIH) high CD200R+ monocyte numbers at baseline that further increased following IBT. Recently, CD200R was described as dampening the production of inflammatory cytokines by myeloid cells in healthy people. Nevertheless, in IFN-alpha-mediated inflammation, CD200R can amplify the immune response (51). Type I interferon activation is also described for AIH (52), and it is likely that IBT triggers the induction of IFN-alpha. With regard to immunotherapies and their increasing use in combination with local ablative therapies, the CD200–CD200R axis is of major importance and additional investigations are needed to identify targets harmonizing combinations of both therapy types.

In summary, local ablation by IBT and RFA causes an early systemic innate immune response and modulates myeloid cell populations in the peripheral blood of HCC patients. We demonstrate that IBT leads to changes in levels of several monocyte subpopulations as well as mMDSC and significantly alters expression levels of myeloid markers whereas RFA does less. Future studies are necessary to explore the impact of the therapy-induced innate immune response on tumor cells and how myeloid-targeting immunotherapies could be combined with interventional strategies. Regardless of mechanism, we hypothesize that radiation-based therapy may be more advantageous when combined with immune-oncology. Thus, in the future, with further validation, addressing myeloid cells and their function may become an adjunct to alter effects of local ablative therapies.

## Data availability statement

The datasets generated during and/or analyzed during the current study are available from the corresponding author on reasonable request.

## Ethics statement

This study was reviewed and approved by Ethics commission of the University hospital, LMU München, Munich, Germany. The patients/participants provided their written informed consent to participate in this study.

## Author contributions

MK, SK, LR, MRS, and MW conceived the experiments. MK, SK, EÖ, MS, and MW carried out the experiments. MK, SK, EÖ, MÜ, OÖ, MRS, MS, and MW analyzed the data. MK, SK, and MW wrote the manuscript. All authors contributed to manuscript revision, read, and approved the submitted version.

## Acknowledgments

We would like to thank the Study Center (Department of Radiology, University Hospital, LMU Munich) for the study management and Cheryl Gray for technical assistance.

## Conflict of interest

The authors declare that the research was conducted in the absence of any commercial or financial relationships that could be construed as a potential conflict of interest.

## Publisher’s note

All claims expressed in this article are solely those of the authors and do not necessarily represent those of their affiliated organizations, or those of the publisher, the editors and the reviewers. Any product that may be evaluated in this article, or claim that may be made by its manufacturer, is not guaranteed or endorsed by the publisher.
