# Supplementary material for: Early monocyte response following local ablation in hepatocellular carcinoma
Source: Front Oncol. 2022 Oct 24;12:959987. doi: 10.3389/fonc.2022.959987 (PMC9638411; doi:10.3389/fonc.2022.959987)
Supplement: Supplementary file 1 [file DataSheet_1.pdf]

Supplementary Figure 1

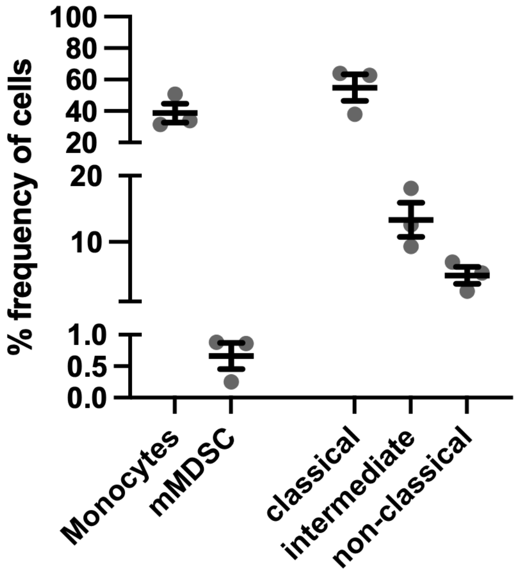

	% cells (Mean ± SD)	*p* value
**Monocytes**	38.70	0.2079 [a]
**mMDSC**	0.66	0.8998 [b]
**classical**	54.87	0.2873 [a]
**intermediate**	13.34	0.4095 [b]
**non-classical**	4.94	0.7818 [b]

Supplementary Figure 1. Healthy Donor data of three non-matched individuals. Monocytes and mMDSC are shown as % of life cells, monocyte subpopulations as % of monocytes. a: One-way ANOVA, b: Kruskal-Wallis test. *P* values < 0.05 indicate statistical significance.

Supplemental Table 1. Characterization of myeloid cells following IBT and RFA.

	Pre IBT	Post IBT	*p*	Pre RFA	Post RFA	*p*
**LMR**	2.06 (2.02)	1.85 ± 1.12	0.0024_b	1.95 ± 0.49	0.19 ± 0.04	$<0.0001_a$
**NMR**	7.70 ± 2.44	10.16 ± 3.56	0.0130_a	6.60 ± 1.20	0.70 ± 0.08	$<0.0001_a$
**NLR**	3.38 ± 1.79	8.26 ± 5.72	0.0044_a	3.57 ± 1.08	3.85 ± 0.99	0.7170_a
**Monocytes (G/µl)**	0.48 (0.09)	0.64 ± 0.22	0.0098_b	0.70 (0.42)	6.66 ± 2.09	0.0078_b
**Lymphocytes (G/µl)**	1.44 ± 0.61	1.14 ± 0.71	0.0486_a	1.19 ± 0.44	1.18 ± 0.40	0.9082_a
**Neutrophils (G/µl)**	3.87 ± 0.75	6.03 ± 1.92	0.0059_a	3.85 ± 1.62	4.50 ± 1.72	0.5592_a
**mMDSC (%)**	0.80 (0.81)	2.30 (2.33)	0.0005_b	0.87 ± 0.63	1.32 ± 0.59	0.0606_a
**Classical (%)**	52.09 ± 9.09	65.62 ± 10.66	0.0027_a	50.09 ± 8.18	46.84 ± 12.44	0.3173_a
**Intermediate (%)**	14.35 (6.90)	11.55 (4.93)	0.1294_b	14.50 (4.60)	20.93 ± 7.80	0.2031_b
**Non-Classical**	3.74 (4.62)	1.01 (0.79)	0.0005_b	4.67 ± 2.84	2.86 ± 2.02	0.0088_a
**CD86+**	86.20± 6.16	81.25 ± 8.25	0.0772_a	81.82 ± 5.54	71.50 ± 11.86	0.0508_a
**CD163+**	55.02 ± 11.92	80.18 ± 10.78	$<0.0001_a$	49.46 ± 11.63	62.36 ± 18.51	0.1298_a
**CD200R+**	15.95 (14.03)	10.40 (13.15)	0.0425_b	16.70 (39.18)	15.90 (34.9)	0.3594_b

Values are depicted as mean (± SD) or median (IQR). a: paired t-test, b: Wilcoxon test.
P values < 0.05 indicate statistical significance
